# Correction: Exploring the relationship between motor visual proficiency and performance metrics in elite skeet shooters: An in-depth analysis

**DOI:** 10.1371/journal.pone.0340299

**Published:** 2026-01-05

**Authors:** Dongxu Gao, Beishi Hu, Tinggang Yuan, Qingshou Guo, Pengfei Wei, Yang Wu, Chao Chen

The authors, Yang Wu and Tinggang Yuan, should be noted as shared co-corresponding authors on this work. Tinggang Yuan’s email address is: yuantinggang@ciss.cn.
