# Supplementary material for: Exploring the relationship between motor visual proficiency and performance metrics in elite skeet shooters: An in-depth analysis
Source: PLoS One. 2025 Jun 2;20(6):e0325351. doi: 10.1371/journal.pone.0325351 (PMC12129156; doi:10.1371/journal.pone.0325351)
Supplement: S1 Appendix — (DOCX) [file pone.0325351.s001.docx]

**S1: Description of Motor Visual Test Indicators of Chinese National Skeet Shooters**

| Test items | Basic Position and Starting Posture | Test Purpose | Specifics and Requirements |
| --- | --- | --- | --- |
| visual clarity (VC)  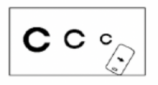 | Subjects stood at a distance of 3m from the tablet with the mobile in hand. | The task tests visual acuity for details at a given distance. Reflect the athlete's static visual acuity. | The athletes were tasked with determining the direction of the notch within the C-shaped pattern displayed on a flat panel screen and subsequently sliding the mobile end in the corresponding direction. Initially, the athletes' left and right monocular vision were assessed, followed by an evaluation of their binocular vision. |
| Contrast Sensitivity (CS)  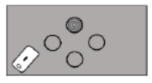 | Subjects stood at a distance of 3m from the tablet with the mobile in hand. | A metric was employed to assess the | Four black circles are presented on the plate, with one of the circles containing concentric circles of varying shades oriented in a random direction. The subject is required to identify the direction of these concentric circles and subsequently slide the moving end in the corresponding direction. |
|  |  | minimum resolvable difference in contrast at a specified distance, which serves as an indication of the athlete's visual system's capacity to process the target object and its background space under diverse luminance conditions. |  |
| Depth of Perception (DP)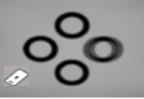 | Subjects stood at a distance of 3m from the tablet with the mobile in hand. | The assessment evaluates the speed and accuracy of subjects in detecting variations in depth at a fixed distance while utilizing LCD glasses. This measurement reflects the athlete's proficiency in rapidly and precisely estimating the distance of objects in their line of sight and understanding the spatial positional relationships. | Four black circles are displayed on the screen, with one of them containing a stereoscopic image. The athlete is instructed to locate this circle and slide it in the appropriate direction on the mobile device. The assessment begins with a binocular evaluation, followed by separate tests for the right and left eyes. |
| Near/Far Switching (N/F Q)  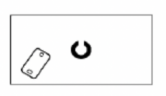 | Subjects were 3m away from the tablet, with the top of the handheld mobile at the bottom of the tablet screen and 40cm from the eyes. | This test measures the number of near and distant targets that can be accurately identified within a 30-second timeframe. It assesses the athlete's regulatory convergence function, which pertains to their capacity to shift visual attention and focus between distant and near objects. | During the test, the Senaptec tablet display (distal) and mobile device (proximal) will alternately present a C pattern. The athlete is required to shift their focus between the distal and proximal stimuli to determine the direction of the notch within a 30-second timeframe. Subsequently, they must rapidly slide the mobile device in the corresponding direction. |
| Perceived Range (PS)  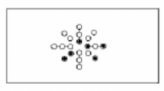 | The athlete stands 60cm from the tablet at eye level with the centre of the screen. | This assessment measures the subject's capacity to retain and reconstruct visual patterns. It evaluates the athlete's visual perceptual clay targetrimination by employing dual visual fields to gauge object recognition, speed, and span. | A specific number of circles appear on the screen, emanating from the center, with some of these circles featuring black dots that flash rapidly at their core. The athlete is tasked with identifying the circle containing the flashing black dot and determining the precise location to click. |
| Multi-target Tracking (MOT)  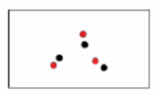 | The athlete stands 60cm from the tablet at eye level with the centre of the screen. | This assessment measures the subject's capacity to track multiple moving targets simultaneously, which reflects various facets of attention, including selective attention, divided attention, and sustained attention. | Upon initiating the test, multiple groups of balls emerged on the screen, with each group comprising two black balls. One of these balls transitioned to red and promptly reverted to black, subsequently commencing a continuous rotation in both clockwise and counterclockwise directions. Following the cessation of rotation, the subject was required to identify the ball within each group that had initially turned red. |
| Response Time (RT)  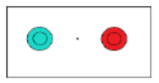 | The athlete stands 60cm from the tablet at eye level with the centre of the screen. | The Simple Reaction Time test serves as an indication of the athlete's reaction speed and responsiveness to basic visual stimuli, quantifying the duration required for a subject to initiate a motor response upon receiving a simple stimulus signal. | The participant initially selects their dominant hand. Subsequently, two circles are presented on the screen. By touching the center of the circles with the index fingers of both hands, the circles will change color to green. The circles will then transition to red in a randomized manner, prompting the participant to rapidly raise and lower the corresponding index fingers. |
| Target Capture (TC)  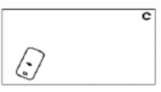 | The subject stands 3m away from the screen with the mobile in hand and adjusts the blue line in the centre of the screen to be at eye level. | This assessment measures the velocity with which subjects shift their attention and recognize targets in the peripheral visual field, thereby reflecting the athlete's capacity for visual saccadic tracking. | The subjects fixated their gaze on the center of the large screen. Subsequently, a C pattern would abruptly and randomly emerge in one of the four corners of the screen. The subjects were required to assess the direction of the gap and mark the corresponding direction on the moving end. |
| Hand-eye Coordination (EHC)  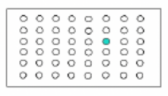 | The athlete stands 60cm from the large screen and raises the centre line of the screen at arm's length or slightly higher so as not to affect the speed of touching the target in the peripheral field of view. | This assessment quantifies the subject's hand speed in response to a rapidly changing target guided by visual orientation. It is predicated on the acquisition, processing, and integration of visual information to direct and regulate bodily movements in a rapid, coherent, and precise manner. This measurement reflects the collaborative capacity of the visual system and the motor execution system. | A large display is presented, consisting of 8 columns and 10 rows of hollow circles. During the test, one of these circles randomly changes color to blue-green, and upon the subject clicking on it, another circle appears at a random location. The objective of the test is for the subject to click on as many circles as possible within the given time frame, while maintaining both speed and accuracy. |
| Decision-Making Mechanisms (G/NG)  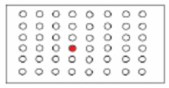 | The athlete stands 60cm from the large screen and raises the centre line of the screen at arm's length or slightly higher so as not to affect the speed of touching the target in the peripheral field of view. | This assessment measures the degree of execution and inhibition of visually guided hand responses in response to Go and No-Go stimuli. It serves as an indication of the athlete's ability to regulate their own response inhibition and cognitive flexibility, which is based on the rapid and accurate identification of the target. | The Senaptec large screen will display the same configuration of 8 columns of circles as used in the eye-hand coordination test. Random green or red dots will appear on the screen; the athlete is instructed to tap quickly when a green dot appears, while refraining from tapping when a red dot is displayed. |

Note: Each test was conducted only once.
