# Supplementary material for: Exploring the relationship between motor visual proficiency and performance metrics in elite skeet shooters: An in-depth analysis
Source: PLoS One. 2025 Jun 2;20(6):e0325351. doi: 10.1371/journal.pone.0325351 (PMC12129156; doi:10.1371/journal.pone.0325351)
Supplement: S2 Table — Motor Visual Ability Test. (DOCX) [file pone.0325351.s002.docx]

S2: Specialized Scores and the Indicators of the Basic Motor Visual Ability Test

| **Test Contents** | **Test metrics** | **Results** | **95%CI** | |
| --- | --- | --- | --- | --- |
|  |  |  | **Lower Limits** | **Higher Limits** |
| Average of Results | Average of specialized results in the three World Cup selection races | 112.37±4.69 | 111.73 | 113.00 |
| Visual Clarity (VC) | VC_R (5-point scale) the closer to 5.0 the better | 4.83±0.23 | 4.797 | 4.859 |
|  | VC_L (5-point scale) the closer to 5.0 the better | 4.87±0.08 | 4.855 | 4.876 |
|  | VC_B (5-point scale) the closer to 5.0 the better | 4.96±0.04 | 4.955 | 4.966 |
| Contrast Sensitivity (CS) | CS_6/logCS | 2.00±0.02 | 1.997 | 2.002 |
|  | CS_18/logCS | 1.98±0.12 | 1.964 | 1.996 |
| Depth of Perception (DP) | DP_P/arcsec | 106.08±14.13 | 104.152 | 107.998 |
|  | DP_L/arcsec | 131.55±10.98 | 130.059 | 133.045 |
|  | DP_R/arcsec | 126.34±15.25 | 124.264 | 128.412 |
| Near/Far Switching (N/FQ) | NFQ_SCORE | 24.57±0.89 | 24.450 | 24.692 |
|  | NFQ_N_RT/ms | 882.86±20.55 | 880.064 | 885.654 |
|  | NFQ_F_RT/ms | 1214.52±9.99 | 1213.159 | 1215.876 |
| Target Capture (TC) | TC /ms | 231.73±9.21 | 230.480 | 232.987 |
| Perceived Range (PS) | PS/Each | 67.44±22.51 | 64.381 | 70.505 |
| Multi-Target Tracking (MOT) | MOT_P_S | 0.76±0.04 | 0.753 | 0.764 |
|  | MOT_C_S | 1849.18±552.23 | 1774.052 | 1924.301 |
|  | MOT_OBJ/pc | 5.00±0.40 | 4.951 | 5.059 |
|  | MOT_SPEED/（degrees/s） | 479.41±95.65 | 466.403 | 492.426 |
| Hand-Eye Coordination (EHC) | EHC_T/ms | 47140.84±2489.72 | 46802.141 | 47479.533 |
|  | EHC_RT/ms | 597.91±24.12 | 594.629 | 601.192 |
|  | EHC_C_RT/ms | 536.02±46.92 | 529.641 | 542.406 |
|  | EHC_P_RT/ms | 622.23±42.21 | 616.485 | 627.970 |
| Decision  Making Mechanisms (G/NG) | GNG__SCORE | 8.19±1.03 | 8.046 | 8.325 |
|  | GNG_G_HIT/pc | 7.70±1.14 | 7.540 | 7.850 |
|  | GNG_R_HIT/pc | 0.14±0.8 | 0.092 | 0.194 |
| Response Time (RT) | RT_A/ms | 311.24±1.93 | 310.976 | 311.501 |
|  | RT__D/ms | 306.64±14.65 | 304.646 | 308.631 |
|  | RT_ND/ms | 308.76±33.22 | 304.239 | 313.276 |

*Note: VC_R. right eye visual acuity; VC_L. left eye visual acuity; VC_P. binocular visual acuity; CS_6 spatial frequency contrast thresholds for 6-week bars per degree of view, CS_18 spatial frequency contrast thresholds for 18-week bars per degree of view; DP_P binocular depth-of-perception thresholds, DP_L left depth-of-perception thresholds, DP_R right depth-of-perception thresholds; NFQ_SCORE near/far switching 30s score; NFQ_N_RT far-near switching proximal average reaction time, NFQ_F_RT far-near switching distal reaction time; TC target capture limiting speed; PS perceptual range number; MOT_P_S multi-target tracking proportional score; MOT_C_S multi-target tracking composite score; MOT_OBJ multi-target tracking highest trackable number; MOT_SPEED multi-target tracking highest trackable speed; MOT_SPEED multi-target tracking highest trackable speed; MOT_SCORE multi-target tracking highest trackable speed; MOT_SCORE Target Tracking Maximum Trackable Speed, EHC_T Hand-Eye Coordination Overall Time Used, EHC_RT Hand-Eye Coordination Average Reaction Time; EHC_C_RT Hand-Eye Coordination Centre of View Average Reaction Time; EHC_P_RT Hand-Eye Coordination Peripheral Area Average Reaction Time, GNG_SCORE Decision Mechanism Overall Score, GNG_G Decision Mechanism Number of Correct Clicks, GNG_R Decision Mechanism number of incorrect clicks; RT_A average response time, RT_D dominant hand response time, RT_ND non-dominant hand response time.*
