# Supplementary material for: Exploring the relationship between motor visual proficiency and performance metrics in elite skeet shooters: An in-depth analysis
Source: PLoS One. 2025 Jun 2;20(6):e0325351. doi: 10.1371/journal.pone.0325351 (PMC12129156; doi:10.1371/journal.pone.0325351)
Supplement: S3 Table — (DOCX) [file pone.0325351.s003.docx]

S3: Indicators of the Specialized Visual Ability Test

| Test Contents | Test Purposes | Results | 95%CI | |
| --- | --- | --- | --- | --- |
|  |  |  | Lower Limits | Higher Limits |
| Focus Duration (FD) | FD_HS/s | 1.22±0.14 | 1.201 | 1.238 |
|  | FD_LS/s | 1.25±0.11 | 1.230 | 1.261 |
|  | FD_HD/s | 1.28±0.14 | 1.258 | 1.297 |
|  | FD_LD/s | 1.27±0.13 | 1.256 | 1.291 |
| Focus Count (FC) | FC_HS/Time | 3.51±0.71 | 3.415 | 3.608 |
|  | FC_LS/Time | 1.16±0.33 | 1.111 | 1.200 |
|  | FC_HD/Time | 1.54±0.46 | 1.475 | 1.601 |
|  | FC_LD/Time | 7.39±0.59 | 7.305 | 7.465 |
| Average Eye Jump (ASA) | ASA_HS/px | 221.54±36.05 | 216.637 | 226.447 |
|  | ASA_LS/px | 211.99±31.10 | 207.757 | 216.217 |
|  | ASA_HD/px | 221.73±36.67 | 216.738 | 226.717 |
|  | ASA_LD/px | 221.75±31.77 | 217.424 | 226.068 |
| Pupil Diameter (PD) | PD_HS/mm | 2.90±0.52 | 2.834 | 2.974 |
|  | PD_LS/mm | 2.96±0.57 | 2.884 | 3.038 |
|  | PD_HD/mm | 3.03±0.52 | 2.959 | 3.102 |
|  | PD_LD/mm | 3.09±0.57 | 3.013 | 3.168 |
| Number of Blinks (BC) | BC_HS/Time | 1.12±0.32 | 1.075 | 1.163 |
|  | BC_LS/Time | 1.15±0.31 | 1.110 | 1.195 |
|  | BC_HD/Time | 1.50±0.45 | 1.433 | 1.557 |
|  | BC_LD/Time | 1.50±0.48 | 1.437 | 1.566 |
| Response Time (RT) | RT_HS/ms | 4686.96±202.00 | 4659.479 | 4714.439 |
|  | RT_LS/ms | 4662.95±192.70 | 4636.738 | 4689.167 |
|  | RT_HD/ms | 4708.39±176.94 | 4684.324 | 4732.465 |
|  | RT_LD/ms | 4700.29±211.94 | 4671.460 | 4729.123 |

*Note: FD_HS. high-table single-target gaze duration, FD_LS low-table single-target gaze duration, FD_HD high-table dual-target gaze duration, FD_LD low-table dual-target gaze duration; FC_HS. high-table single-target gaze count, FC_LS low-table single-target gaze count, FC_HD high-table dual-target gaze count, FC_LD low-table dual-target gaze count; ASA_HS. high-table single-target mean ocular amplitude, ASA_LS low-table dual-target mean ocular amplitude, ASA_LD low-target dual-target mean ocular amplitude; ASA_LS. Average eye-beat amplitude for single target on high platform, average eye-beat amplitude for single target on low platform in ASA_LS, average eye-beat amplitude for double target on high platform in ASA_HD, average eye-beat amplitude for double target on low platform in ASA_LD; PD_HS. Pupil diameter of single target on high platform, pupil diameter of single target on low platform in PD_LS, pupil diameter of double target on high platform in PD_HD, pupil diameter of double target on low platform in PD_LD; BC_HS. number of low-table single-target blinks, BC_HD number of high-table double-target blinks, BC_LD number of low-table double-target blinks; RT_HS. number of high-table single-target responses, RT_LS number of low-table single-target responses, RT_HD number of high-table double-target responses, RT_LD number of low-table double-target responses.*
