# Supplementary material for: Exploring the relationship between motor visual proficiency and performance metrics in elite skeet shooters: An in-depth analysis
Source: PLoS One. 2025 Jun 2;20(6):e0325351. doi: 10.1371/journal.pone.0325351 (PMC12129156; doi:10.1371/journal.pone.0325351)
Supplement: Attachment — Submitted filename: Response to Reviewers.docx [file pone.0325351.s006.docx]

PONE-D-24-54464R1: PLOS ONER1

Title: **Exploring the Relationship between Motor Visual Proficiency and Performance Metrics in Elite Skeet Shooters: An In-depth Analysis**

Article Type: **Research Article**

Dear Editor

**Response to the review comments:**

Thank you for the useful comments and suggestions on our manuscript. We have included required information and revised the manuscript accordingly, and detailed corrections are listed below:

**Response to comments by Academic Editor:**

1. Please ensure that your manuscript meets PLOS ONE's style requirements, including those for file naming.

*We have rechecked and corrected the mistakes as suggestion given.*

2. Thank you for stating the following financial disclosure: “Funding Dongxu Gao and Tinggangyuan China Institute of Sport Science Basic Scientific Research Business Funding Project,(Basic 23-39)” Please state what role the funders took in the study. If the funders had no role, please state: "The funders had no role in study design, data collection and analysis, decision to publish, or preparation of the manuscript.

"*We have added the funding information as suggestion given”*

*{Please refer to highlights in the cover letter]*

3. Thank you for stating the following in your Competing Interests section:
   “The authors have stated that there is no competing interest.” Please complete your Competing Interests on the online submission form to state any Competing Interests. If you have no competing interests, please state "The authors have declared that no competing interests exist.", as detailed online in our guide for authors at <http://journals.plos.org/plosone/s/submit-now>. This information should be included in your cover letter; we will change the online submission form on your behalf.

"*We have added the competing interest information as suggestion given”*

*{Please refer to highlights in the cover letter]*

4. PLOS requires an ORCID iD for the corresponding author in Editorial Manager on papers submitted after December 6th, 2016. Please ensure that you have an ORCID iD and that it is validated in Editorial Manager.

*“The corresponding author has added ORCID in the submission account as suggestion given.”*

5. Please include captions for your Supporting Information files at the end of your manuscript, and update any in-text citations to match accordingly.

*We have included captions for supporting information files at the end of the manuscript as suggested.*

*[Please refer to highlights at the end of the manuscript]*

6. We note that Figures 3 and 5 includes an image of participants.

*We have covered the identifiable parts of the study participants in figure 3 and 5.*

*[Please refer to figure 3 and figure 5]*

Sincerely

Yang Wu
