# Supplementary material for: Exploring the relationship between motor visual proficiency and performance metrics in elite skeet shooters: An in-depth analysis
Source: PLoS One. 2025 Jun 2;20(6):e0325351. doi: 10.1371/journal.pone.0325351 (PMC12129156; doi:10.1371/journal.pone.0325351)
Supplement: Attachment — Submitted filename: Response_to_Reviewers_auresp_2.docx [file pone.0325351.s007.docx]

PONE-D-24-54464R2: PLOS ONER2

Title: **Exploring the Relationship between Motor Visual Proficiency and Performance Metrics in Elite Skeet Shooters: An In-depth Analysis**

**Short title:** Motor Visual Skills and Performance in Elite Skeet Shooters

Article Type: **Research Article**

Dear Editor

**Response to the review comments:**

Thank you for the useful comments and suggestions on our manuscript. We have included required information and revised the manuscript accordingly, and detailed corrections are listed below:

**Response to comments by Academic Editor:**

1. The references still are not uniform. Please follow the instructions to authors in uniformly formatting all of the references. I won't be able to look for reviewers until this matter is resolved.

Dear editor, thank you for your feedback on references. We have rechecked and amended the entire references and citations based on the instructions to authors as suggested.

[Please refer to highlights in the reference section]

2. Please upload your figure files to the Preflight Analysis and Conversion Engine (PACE) digital diagnostic tool.

We have uploaded all figures to PACE tool, all figures were improved accordingly.

Sincerely

Yang Wu
