# Supplementary material for: Exploring the relationship between motor visual proficiency and performance metrics in elite skeet shooters: An in-depth analysis
Source: PLoS One. 2025 Jun 2;20(6):e0325351. doi: 10.1371/journal.pone.0325351 (PMC12129156; doi:10.1371/journal.pone.0325351)
Supplement: Attachment — Submitted filename: Response_to_Reviewers_auresp_3.docx [file pone.0325351.s008.docx]

PONE-D-24-54464R2: PLOS ONER3

Title: **Exploring the Relationship between Motor Visual Proficiency and Performance Metrics in Elite Skeet Shooters: An In-depth Analysis**

**Short title:** Motor Visual Skills and Performance in Elite Skeet Shooters

Article Type: **Research Article**

Dear Editor

**Response to the review comments**

Thank you for the useful comments and suggestions on our manuscript. We have included the required information and revised the manuscript accordingly, and a point-by-point response to review questions is listed below:

**Response to journal requirements**

1. Please review your reference list to ensure that it is complete and correct. If you have cited papers that have been retracted, please include the rationale in the manuscript text or remove these references and replace them with relevant current references. The rebuttal letter should mention any changes to the reference list that accompany your revised manuscript. If you need to cite a retracted article, indicate the article's retracted status in the References list and include a citation and full reference for the retraction notice.

**Response:** *We have checked all references for retraction and completeness, and no retracted articles were identified on the list. All references are currently valid as per Retraction Watch and PubMed checks. We have also replaced some blogs and duplicate references with recent empirical studies.*

**Response to comments by Academic Editor**

1. Please comment on reviewer #1's comments regarding the statistical analysis. While the overall r-squared value is quite good, the correlation coefficients between various tests are somewhat low. Please comment on the implications of these lower correlations. Reviewer #1 also asks for some details about the aSee glasses. Presumably, these glasses are independent of the Senaptec system. If the aSee glasses are not related to the Senaptec system, please indicate this.

**Response:** *Dear editor, Thank you for your overall feedback. We have addressed reviewer 1's comments. The detailed point-by-point response is mentioned under response to reviewer 1 comments below and highlighted in the method, results, and discussion section of the manuscript.*

2. Please provide the manufacturer of the aSee system, the resolution (which looks like 0.50deg from the company website (https://www.7invensun.com/elite)), and, if known, the accuracy of this eye tracking system.

**Response:** *Thank you for requesting specification details. We have addressed this comment and provided the details regarding the aSee eye-tracking system, as suggested.*

Please refer to highlights in the method section of the manuscript where we have addressed this comment.

*[…The specialized visual ability test utilized the aSee Glasses (7INVENSUN, Beijing, China), a spectacle-type eye-tracking device with a resolution of 0.50° and an accuracy of 0.5°–1.0°. The system’s high-frequency sampling rate (120 Hz) and precision make it suitable for capturing dynamic gaze behaviors in sport-specific scenarios….]*

**Response to comments by Reviewer #1**

1. I don't understand statistics about r because it is relatively low.

# Response: *Thank you for this insightful clarification question and comments. While the correlation coefficients (r-values) reported in our study (e.g., 𝑟 =0.486 for Perceived Range) may appear modest, they are consistent with findings in sports science and human performance research, where multifactorial influences (e.g., psychological, physiological, environmental) often result a significant effect on performanes. Even moderate correlations (e.g., r=0.3–0.5) are meaningful in such contexts, reflecting measurable and actionable relationships between variables. For example, r=0.486 (Perceived Range vs. performance) explains ~23.6% of the variance (𝑅^2^ =0.237), which is substantial in elite athlete populations where marginal gains are critical.*

[Please refer to highlights in the discussion part where we have addressed this comment]

2. I like the Senaptec system but do not see the relation with aSee Glasses. Can you explain a little more?

**Response:** *The Senaptec system assesses basic motor visual abilities (e.g., visual clarity, depth perception, reaction time) under controlled conditions, providing foundational metrics of athletes’ visual hardware. In contrast, the aSee Glasses evaluate specialized visual abilities (e.g., gaze patterns, target tracking, decision-making) during dynamic, sport-specific tasks, reflecting how athletes apply these foundational skills in ecologically valid scenarios. Together, they provide a comprehensive evaluation: Senaptec identifies baseline visual capabilities, while aSee Glasses reveals how these capabilities translate to performance under competition-like demands.*

[Please refer to the highlights in the materials and method section where we explained this comment.]

Sincerely

Yang Wu
